# Perceptions of a naloxone leave behind program among emergency medical services personnel in Michigan, USA

**DOI:** 10.1016/j.dadr.2024.100273

**Published:** 2024-08-14

**Authors:** Jason Brian Gibbons, Olivia K. Sugarman, Lauren Byrne, Samantha J. Harris, Hridika Shah, Eric G. Hulsey, Julie Rwan, Esther Mae Rosner, Anthony Pantaleo, Emily Bergquist, Brendan Saloner

**Affiliations:** a Department of Health Systems, Management and Policy, Colorado School of Public Health, University of Colorado Anschutz Medical Campus, 13001 E 17th Pl, Aurora, CO 80045, USA; b Department of Health Policy and Management, Bloomberg School of Public Health, Johns, Hopkins University, 624 N. Broadway, Baltimore, MD 21205, USA; c Overdose Prevention Program, Vital Strategies, 100 Broadway, 4th Floor, New York, NY, 10005, USA; d Michigan Department of Health and Human Services, USA

**Keywords:** Naloxone leave behind, Opioid overdose, Emergency medical services

## Abstract

**Introduction:**

In 2020, Michigan implemented its first Naloxone Leave-Behind Program for Emergency Medical Service (EMS) field providers. Under the program, EMS field providers leave naloxone kits to individuals aged 15 or older they encounter in the field who have overdosed, who indicate they have a substance use disorder, or exhibit signs of opioid use and/or to bystanders, friends, or family that are present at the encounter.

**Methods:**

Survey of EMS field providers and administrators to assess perspectives on the Michigan NLB program. Comparisons of perspectives between field providers and administrators working in EMS agencies operating in medical control authorities (MCAs) participating in the NLB program (i.e., participating agencies) with field providers and administrators working for EMS agencies serving non-participating MCAs.

**Results:**

Most EMS field providers and administrators supported the Michigan NLB program. However, some were concerned about the unintended consequences of leaving behind naloxone, including the potential for recipients to use more drugs or be less likely to seek treatment. Perspectives of NLB program effectiveness were similar between EMS administrators and field providers. Participating administrators’ top-cited barrier to implementation was convincing field providers to leave behind naloxone, while non-participating administrators were concerned with stocking naloxone kits.

**Conclusions:**

Additional engagement and training to address concerns by EMS field providers and administrators about the benefits of the NLB program are needed to expand program participation intensity. Streamlining naloxone procurement and increasing messaging about free access to naloxone for participating in the program may help increase adoption.

## Highlights

• Michigan’s EMS providers generally supported the Naloxone Leave Behind program.
• Despite recognition of the program’s potential to save lives, adoption has been low.
• EMS Providers are concerned that the program could lead to unintended consequences.
• Stocking naloxone and getting providers to leave behind kits has been a barrier.
• Mitigating concerns and streamlining naloxone procurement may increase adoption.

## 1 Introduction

In 2019, nearly 1 in every 17 people aged 15–64 used drugs annually, or about 296 million people worldwide (UNODC, 2023), resulting in over 600,000 drug overdose deaths (World Health Organization, 2023). Opioids have been the largest contributor to global drug overdose deaths, and currently account for more than two-thirds of all drug-related fatalities (UNODC, 2023). Opioid overdose deaths are a particularly significant public health challenge in the US, which declared the opioid epidemic a national public health emergency in 2017. In comparison with 17 other high-income countries, US overdose death rates are three and a half times higher (Ho, 2019), which is largely explained by deaths from opioid overdoses. Specifically, in 2022, over 80,000 Americans experienced a fatal opioid overdose, representing nearly 80 % of all drug overdoses in that year (National Center for Health Statistics, 2022). Also, like many other countries, US opioid overdose deaths have accelerated in recent years, rising from 8.2 deaths per 100,000 in 2002–32.6 in 2022 (Spencer et al., 2024).

To reduce rising US opioid overdose deaths, expanding the reach of naloxone, an opioid overdose reversal agent, has become a public health priority. In the US, naloxone access is routinely carried by first responders and has increasingly become available to lay people, including people who use drugs. A recent innovation in the delivery of naloxone has been the development of Naloxone Leave Behind programs (NLBs). In an NLB program, emergency medical service (EMS) providers leave behind a naloxone kit to individuals who have experienced an overdose in the community and are at greater risk of subsequent overdose (Olfson et al., 2018). Several US states have worked to implement NLBs with their EMS providers, including Pennsylvania (Pennsylvania Pressroom, 2019)_,_ California (LeSaint et al., 2022), Maryland (Maryland Department of Health, 2023), and Michigan (Michigan Department of Health and Human Services, 2020).

Since their inception, NLBs have increased naloxone distributions across several major United States cities and counties. For example, a program led by the City of Philadelphia Fire Department in collaboration with the Pennsylvania Department of Health left behind over 2000 naloxone kits for overdose survivors (Pennsylvania Pressroom, 2019). In a program in San Francisco, over 1200 kits were distributed to EMS field providers to leave behind (LeSaint et al., 2022). In Howard County, Maryland, EMS left behind over 7000 kits to nearly 3000 individuals (Maryland Department of Health, 2023). Some studies have started to quantify the effect of NLB programs on population health. One recent study from Maryland showed that individuals who received a naloxone kit from the state’s NLB program were 2.4 times more likely to obtain follow-up treatment services than those who did not receive naloxone (Scharf et al., 2021).

Our study focuses on Michigan, which implemented a statewide NLB program under the direction of the Michigan Department of Health and Human Services (MDHHS) in August 2020 (District Health Department, 2020, Michigan Department of Health and Human Services, 2020). Under the Michigan NLB program, Medical Control Authorities (MCAs) directors decide on a voluntary and rolling basis to have their entire MCA region participate. An MCA is an organization designated by MDHHS to supervise, train, authorize, and coordinate emergency medical services within a particular geographic region in Michigan (Michigan Department of Health and Human Services, 2023a). Each MCA is associated with one of 60 geographic regions that comprise Michigan. MCAs have significant autonomy in overseeing and coordinating EMS within that region, including whether to adopt new EMS-related programs and protocols. The MCA structure in Michigan is a unique regional mechanism of EMS oversight and policy enforcement whereas many other states utilize a statewide approach.

After adopting the MDHHS NLB protocol, EMS agencies operating in the MCA region are informed and instructed to begin training their field providers in NLB protocols. MDHHS offers both online and in-person training options multiple times per year across the state to maximize training opportunities and uptake. The MCA then orders naloxone kits for all EMS agencies operating within the MCA through an MCA pharmacy. The EMS agency picks up the kits from the MCA pharmacy and then creates packages with informational pamphlets provided by MDHHS and local treatment organizations within the MCA. Informational pamphlets include guidance about identifying overdose, how to administer naloxone, and other information on how to respond to an overdose (e.g., open the airway and give rescue breaths, put the overdose victim on their side with their legs and arms crossed to prevent choking, and stay until help arrives). Finally, the EMS field providers disseminate kits during emergency response encounters. The criteria for kit dissemination are encountering individuals who 1) experienced an opioid overdose, 2) indicate substance use disorder, or 3) have signs or symptoms of opioid use. Kits may also be disseminated to patients, family, friends, or bystanders present at the encounter location (Michigan Department of Health and Human Services, 2020).

While early efforts to expand NLB participation by MDHHS have been promising, the degree to which the NLB program is adopted is ultimately shaped by ‘buy-in’ among participating EMS field providers and administrators; preexisting perceptions of naloxone and NLB programs determine program participation intensity. Recent studies have presented mixed findings about EMS field providers' perceptions of naloxone and NLB programs (Hruschak et al., 2020, Martin et al., 2022, Montoy et al., 2022; Tobin et al., 2005). For example, only 13 % of respondents to a 2022 survey of Tennessee EMS providers believed EMS agencies should distribute naloxone to patients and bystanders, and 15.2 % perceived EMS naloxone distribution as a realistic strategy to prevent overdoses (Martin et al., 2022). In contrast, 67 % of San Francisco EMS providers agreed that EMS clinicians should train people to administer naloxone, and 98 % perceived NLB programs as effective in a 2021 survey (Montoy et al., 2022).

Our study summarizes a survey of EMS field providers and administrators that asked about their perceptions of Michigan’s NLB program (e.g., EMS chiefs, other supervisors, and clerical support staff). This analysis aims to improve the NLB program in Michigan and contribute to the general knowledge base of EMS NLB programs. Our findings may also be useful for other US states and countries considering implementing similar programs.

## 2 Materials and methods

### 2.1 Study design

We surveyed Michigan EMS field providers and administrators across all Michigan MCAs (both NLB participating and non-participating MCAs) to measure perceptions and attitudes about naloxone and Michigan’s NLB program and to identify potential differences in perceptions by EMS provider role (i.e., administrator versus field providers). Separate surveys were created for administrators and field providers to gauge their unique perspectives, with overlapping questions to allow for comparisons. Questions from the administrator and field providers' surveys were developed by adapting instruments from naloxone programs in other settings and surveys of general naloxone perceptions among EMS field providers. (Burstein et al., 2020, Hruschak et al., 2020, Martin et al., 2022, Montoy et al., 2022, Peckham et al., 2018, Rudolph et al., 2018; Tobin et al., 2005). See Appendix 1 for the included instruments

### 2.2 Study population

To be eligible to complete the survey, respondents had to be 18 or older, be able to complete the survey in English, and be currently affiliated with a Michigan EMS agency. We sent the survey description and link to participate in the study via the MDHHS EMS weekly email newsletter sent to 40,000 Michigan EMS field provider and administrator email addresses (note that some email addresses may include inactive EMS providers and administrators or duplicate subscribers with multiple email addresses, so we cannot determine the unique number of eligible respondents). Participants from all types of EMS agencies in Michigan (i.e., municipal-owned, fire-based, private, and hospital-owned) were included. There were no restrictions on the number of individuals that could take the survey; multiple individuals at the same EMS agency and role (e.g., field provider or administrator) could participate, conditional on eligibility. Surveys were distributed online from January 2023 to February 2023 using Qualtrics.

A total of 513 EMS providers consented to the online survey. However, 77 were excluded for not completing the survey, 44 were excluded for not being contracted with an EMS agency, and 22 were excluded due to not being active EMS field providers or administrators. EMS field providers were defined as survey respondents who reported their role as a “full-time provider,” “part-time provider,” or “unpaid on-call volunteer” (N=288) and EMS administrators were participants who selected their role as “administrator” (N=82). Our final sample included a total of 370 EMS field providers and administrators.

### 2.3 Statistical analysis

We conducted univariate analyses of survey responses to assess EMS field provider and administrator perspectives on naloxone and the Michigan NLB program by tabulating response options for all single and multi-choice questions. For questions with Likert-scale agreement response options (i.e., strongly disagree, slightly disagree, neither disagree nor agree, slightly agree, strongly agree), we calculated the mean of the responses to determine the net proportion of agreement or disagreement with each survey statement or question. Denominators differed across questions based on the role the question was asked to (e.g., administrator versus field provider) and the respondent's ability to skip questions. Therefore, we report denominators and numerators with all proportions for clarity. We also performed additional response comparisons between EMS field providers and administrators for questions asked of both to understand differences in perspectives between employees and people managing the agencies. P-values were calculated using chi-square tests to determine statistically significant differences in agreement with each item by role (i.e., field provider versus administrator). Finally, we compared the EMS administrator responses to questions about NLB implementation barriers and facilitators between administrators whose agencies were participating in NLB and administrators whose agencies were not participating.

This project was determined to be exempt by the Johns Hopkins Bloomberg School of Public Health Institutional Review Board.

## 3 Results

### 3.1 Characteristics of study participants

Table 1 presents the descriptive characteristics of EMS field providers and administrators participating in the survey. The majority of field providers were licensed as paramedics (n=152/288, 52.7 %) or emergency medical technicians (n=103/288, 35.8 %), and most had received their licenses ten or more years prior (n=182/288, 63.2 %). Administrators and field providers were predominantly male (n=261/369, 70.7 %), over the age of 36 (n=288/370, 71.1 %), White (n=317/368, 86.1 %), and non-Hispanic (n=326/369, 88.3 %).

**Table 1:** Characteristics of Emergency Medical Service (EMS) Field Providers & Administrators Participating in the Naloxone Leave Behind Survey.

**Question**	**Response**	**N**	**%**
**Roles & Licensing**			
What is your primary role? (N=370)	Full-time field provider	186	50.3 %
	Agency administrator	82	22.2 %
	Paid on-call or volunteer provider	65	17.6 %
	Part-time field provider	37	10.0 %
For how many years have you been working in this role? (N=370)	< 1 year	24	6.5 %
	>=1 year and < 2 years	24	6.5 %
	>=2 years and < 5 years	67	18.1 %
	>=5 years and < 10 years	64	17.3 %
	>= 10 years	191	51.6 %
What is your current license level? (N=288)	Paramedic	152	52.8 %
	Emergency medical technician	103	35.8 %
	Medical first responder	22	7.6 %
	Specialist/advanced EMT	8	2.8 %
	Community paramedic	3	1.0 %
How many years ago did you receive your EMS license? (N=368)	< 1 year	13	3.5 %
	>=1 year and < 2 years	14	3.8 %
	>=2 years and < 5 years	39	10.6 %
	>=5 years and < 10 years	50	13.6 %
	>=10 years	252	68.5 %
**Demographics**			
What is your age? (N=370)	18–25	22	5.9 %
	26–35	72	19.5 %
	36–45	95	25.7 %
	46–55	87	23.5 %
	56–65	60	16.2 %
	> 65 years	18	4.9 %
	Prefer not to answer	16	4.3 %
What is your sex? (N=369)	Male	261	70.7 %
	Female	91	24.7 %
	Prefer not to answer	17	4.6 %
What is your race? (N=368)	White	317	86.1 %
	Other/Unknown	46	12.5 %
	Multiracial	3	0.8 %
	Black or African American	2	0.5 %
Are you of Hispanic Latino or Spanish origin? (N=369)	Yes	7	1.9 %
	No	326	88.3 %
	Prefer not to answer	36	9.8 %
**Overdose & Naloxone Experiences**			
Approximately how many opioid-related overdoses have you responded to in the last 30 days? (N=288)	0	158	54.9 %
	1–5	110	38.2 %
	6+	20	6.9 %
Approximately how many naloxone administrations have you performed during opioid-related overdoses in the last 30 days? (N=130)	0	25	19.2 %
	1–5	90	69.2 %
	6+	15	11.5 %
**Naloxone Leave Behind Awareness & Training**			
Has your EMS agency started leaving naloxone behind as part of the MDHHS Naloxone Leave Behind program? (N=369)	Yes	291	78.6 %
	No	69	18.6 %
	Unsure	10	2.7 %
Is your EMS agency participating in the MDHHS Naloxone Leave Behind Program? (N=369)	Yes	112	30.4 %
	No	195	52.8 %
	Unsure	62	16.8 %
If yes, have you received training on how to leave naloxone behind? (N=93)	Yes	85	91.4 %
	Unsure	3	3.2 %
	No	5	5.4 %
If yes, and you also responded to an overdose in the last 30-days, approximately how many take-home Naloxone kits have you provided during and overdose dispatch in the last 30 days? (N=40)	0	30	75.0 %
	1–5	10	25.0 %
	6+	0	0.0 %
If your EMS agency is not leaving behind naloxone as part of the MDHHS Naloxone Leave Behind program, would you like them to? (N=257)”?	Yes	101	39.3 %
	No	54	21.0 %
	Unsure	102	39.7 %

Notes: Author’s analysis of survey response data. Percentages were calculated by dividing the response N by the number of respondents to the question. A total of 370 possible respondents are included. 82/370 are EMS administrators. Administrators are not included in licensure-related questions or overdose and naloxone experiences. Questions asked about naloxone kit distributions are conditional on having encountered one or more overdoses and responding “Yes” to “Is your EMS agency participating in the MDHHS Naloxone Leave Behind Program?” Questions about receiving naloxone leave-behind training are conditional on responding yes to “Is your EMS agency participating in the MDHHS Naloxone Leave Behind Program?” Question about naloxone administration conditional on responding “1–5” or “6+” to “Approximately how many opioid overdoses have you responded to in the last 30-days?”

Over 75 % of field providers and administrators reported having heard of the NLB program (n=291/370, 78.6 %). Slightly less than a third of field providers and administrators reported that their EMS agency was leaving behind naloxone as part of the MDHHS program (n=112/369, 30.4 %). Among field providers indicating their agency was participating, 91.4 % of said that they had received training to leave behind naloxone (n=85/93, 91.4 %. However, among EMS field providers who reported they had been trained and responded to an overdose in the last 30 days, only a quarter (n=10/40, 25.0 %) said that they had left behind one or more naloxone kits.

### 3.2 Naloxone and NLB program perceptions

Table 2 shows the results of questions asked separately to EMS field providers and administrators. Most EMS administrators (n=65/81, 80.2 %) agreed that their staff could successfully identify individuals who could benefit from naloxone kits in the future and felt comfortable having their staff leave behind naloxone kits to people who needed them (n=53/81, 65.4 %). However, EMS administrators expressed some concern that their staff could be held legally liable if adverse events were to happen after leaving behind naloxone (n=34/81, 42.0 %) and were equally as likely to agree versus disagree that the program could create an administrative challenge (agree: n=25/81, 30.9 %; disagree: n=25/81, 30.9 %). Moreover, 29.6 % of administrators were concerned about the additional challenge to their work from participation (n=24/81, 29.6 %).

**Table 2:** Perceptions of Naloxone and Naloxone Leave Behind – Question Asked of EMS Administrators and Field Providers Separately.

**Question Description**	**Agree**		**Disagree**	
	**N**	**%**	**N**	**%**
**EMS Administrators**				
**How comfortable are you in having your staff provide Naloxone to people who might need it?**				
I am comfortable in having my staff provide naloxone kits	53	65.4 %	8	9.9 %
I am confident in my staffs’ ability to identify people that may benefit from having a naloxone kit	65	80.2 %	3	3.7 %
**What are your perceptions about the impact of an EMS-based leave-behind Naloxone program on EMS agencies?**				
I am worried that my staff will be held legally liable for leaving behind Naloxone	34	42.0 %	29	35.8 %
Naloxone leave-behind programs create significant administrative challenges	25	30.9 %	25	30.9 %
Naloxone leave-behind programs put financial stress on my agency	17	21.0 %	34	42.0 %
Participating in the program would add a challenge to my work	24	29.6 %	29	35.8 %
				
**EMS Field Providers**				
**What are your perceptions about the impact of an EMS-based leave-behind Naloxone program on your work?**				
I am worried that I will be held legally liable for leaving behind Naloxone	82	28.5 %	136	47.2 %
Leaving behind naloxone allows me to do more for the community	149	51.7 %	54	18.8 %
Leaving behind naloxone would be an added challenge to my work	36	12.5 %	168	58.3 %

Notes: Author’s analysis of survey response data. Agree reflects agreement aggregated from “Agree” and “Strongly Agree” survey responses. Disagree reflects disagreement aggregated from “Disagree” and “Strongly Disagree” survey responses. Response totals and proportions from “Neither Agree nor Disagree” are omitted. Responding to questions was optional in all cases; not all questions have complete response rates.

Regarding EMS field providers, 51.7 % agreed that leaving behind naloxone helped them do more for their community (n=149/288, 51.7 %), and only 12.5 % of providers believed that leaving behind naloxone would add a challenge to their work (n=36/288, 12.5 %). However, over a quarter of field providers were concerned that they could be held legally liable for adverse events after leaving behind naloxone (n=82/288, 28.5 %).

Table 3 shows responses by both EMS administrators and field providers to questions about their perceptions of naloxone. Slightly more administrators and field providers disagreed than agreed that naloxone may increase the likelihood of overdose because of a lack of fear about the risk of death (disagree: n=134/369, 36.3 %; agree: n=125/369, 33.9 %). Similarly, they were more likely to disagree than agree that naloxone condones drug use (disagree: n=146/369, 39.6 %; agree: n=114/369, 30.9 %). The vast majority disagreed that naloxone could help address the problem of addiction (n=297/369, 80.5 %).

**Table 3:** Perceptions of Naloxone and Naloxone Leave Behind – Question Asked of both EMS Administrators and Field Providers.

**Question Description**	**Agree**		**Disagree**	
	**N**	**%**	**N**	**%**
**What are your perceptions about Naloxone in general?**				
Naloxone…				
Increases the rate of overdose because of a lack of fear about fatal overdose	134	36.3 %	125	33.9 %
Condones drug use	114	30.9 %	146	39.6 %
Does not address the problem of addiction	297	80.5 %	29	7.9 %
Should only be administered by medical professionals	59	16.0 %	207	56.1 %
			
**What are your perceptions about leaving behind Naloxone and the role of EMS?**				
EMS could have a significant public health impact by leaving behind Naloxone	205	55.6 %	71	19.2 %
EMS have a role to play in overdose prevention	165	44.7 %	90	24.4 %
Leaving behind Naloxone frees up EMS to respond to other emergencies	42	11.4 %	211	57.2 %
Leaving behind Naloxone allows EMS to do more for their community	197	53.4 %	85	23.0 %
Leaving behind naloxone is helpful for any drug overdose (e.g. stimulants)	62	16.9 %	215	58.6 %
Leaving behind naloxone would save lives	221	60.1 %	46	12.5 %
Leaving behind naloxone will encourage people to use even more opioids	128	34.8 %	111	30.2 %
Leaving behind naloxone is a waste of time and money	66	17.9 %	179	48.6 %
**What are your thoughts or perceptions of your EMS agency’s attitudes and resources to support a leave-behind naloxone program?**				
My EMS agency believes that leaving behind Naloxone can help save lives	170	46.1 %	30	8.1 %
My EMS agency encourages us regularly to leave behind Naloxone	77	21.1 %	105	28.8 %
My EMS agency has been supportive of training providers to leave-behind Naloxone	124	33.7 %	60	16.3 %
There is adequate support from my EMS agency to expand program participation	141	38.2 %	58	15.7 %
**What concerns do you have about an EMS-based leave-behind Naloxone program?**				
I am concerned about individuals managing the side effects of naloxone alone	172	46.7 %	98	26.6 %
I am concerned that leaving behind naloxone will lead to unintended consequences	116	31.5 %	131	35.6 %
I am concerned that people who receive naloxone will be less likely to seek treatment	201	54.5 %	90	24.4 %
**Leaving Naloxone behind to people who overdose will be effective in …**				
Increasing utilization of drug treatment programs	50	13.7 %	205	56.0 %
Reducing 911 calls	64	17.5 %	188	51.4 %
Reducing drug overdose events	103	28.2 %	165	45.2 %
Reducing drug use	19	5.2 %	245	67.1 %
Reducing overdose deaths	241	65.7 %	65	17.7 %

Notes: Author’s analysis of survey response data. Agree reflects agreement aggregated from “Agree” and “Strongly Agree” survey responses. Disagree reflects disagreement aggregated from “Disagree” and “Strongly Disagree” survey responses. Response totals and proportions from “Neither Agree nor Disagree” are omitted. Responding to questions was optional in all cases; not all questions have complete response rates.

Table 3 also displays responses by EMS administrators and field providers about the role of EMS in leaving behind naloxone. EMS field providers and administrators tended to agree that EMS could create a significant public health impact by leaving behind naloxone (n=205/369, 55.6 %;), that EMS had a role to play in overdose prevention through leaving behind naloxone kits (n=165/369, 44.7 %) and that leaving behind naloxone allowed them to do more for their community (n=197/369, 53.4 %) and could save lives (n=221/368, 60.1 %). Moreover, 48.6 % of field providers and administrators disagreed that NLB programs were a waste of time and money (n=179/368, 48.6 %).

Respondents also expressed concerns about NLB (see Table 3). EMS field providers' and administrators' biggest concern was that people who use opioids will be less likely to seek out treatment if they have access to naloxone (n=201/370, 54.5 %). Many administrators and field providers were also concerned about the ability of individuals who received leave-behind naloxone kits to manage the side effects of naloxone on their own (n=172/368, 46.7 %). However, the number of respondents who said that they were concerned that leaving behind naloxone to friends and family members of someone who has overdosed could lead to unintended consequences was similar to the number of respondents who said that they weren’t concerned (agree: n=116/368, 31.5 %; disagree n=131/368, 35.6 %). Many providers and administrators were also concerned that providing a naloxone kit could encourage a person to use even more opioids (agree n=128/368, 34.8 %; disagree: n=111/368, 30.2 %) and felt that the NLB program would not free up EMS to respond to other emergencies (n=211/369, 57.2 %). Despite these concerns, most respondents agreed that NLB would be effective in reducing overdose deaths (n=241/367, 65.7 %) (see Table 3).

Respondents were also asked how their agency perceived naloxone and NLB (see Table 3). EMS field providers and administrators perceived that their agencies supported the belief that leaving behind naloxone can save lives (n=170/369, 46.1 %), supported training providers to leave behind naloxone (n=124/369, 33.7 %), and were in favor of expanding the use of leave behind naloxone (n=141/369, 38.2 %). However, EMS administrators and field providers were more likely to disagree than agree that their agencies regularly encouraged them to leave behind naloxone (disagree: n=105/366, 28.7 %; agree: n=77/366, 21.1 %).

### 3.3 Comparing administrator and field provider perspectives of naloxone and NLB

In Appendix Table 1, we compare perceptions of naloxone and naloxone leave behind described in Table 3 between EMS administrators and EMS field providers to understand the potential variation between the perspectives of those in the field versus those in charge of running the EMS agencies. Relative to EMS field providers, EMS administrators were more likely to agree that their agency supported the belief that leaving behind naloxone could save lives (EMS administrator agree: n=46/81, 56.8 %; EMS field provider agree: 124/288, 43.1 %; P=0.028). Further, EMS field providers were more inclined to believe that naloxone could reduce 911 calls than administrators (EMS administrator agree: n=6/81, 7.4 %; EMS field provider agree: 58/284, 20.4 %; P=0.048).

### 3.4 Barriers and facilitators to NLB participation – comparing administrators in agencies participating in NLB against non-participating administrators

Table 4 compares the top barriers and facilitators to NLB participation between EMS administrators working at an EMS agency participating in NLB and administrators working at a non-participating EMS agency. The top three barriers reported by administrators at participating agencies were convincing providers to leave behind naloxone (n=8/13, 61.5 %), training providers how to code leaving behind naloxone (n=6/13, 46.2 %), and provider stigma against naloxone (n=5/13, 38.5 %). The top three barriers reported by administrators working at non-participating agencies were stocking naloxone kits (n=38/54, 70.4 %), financial challenge (n=31/54, 57.4 %), and convincing providers to leave behind naloxone (n=25/54, 46.3 %). The top three facilitators of administrators at NLB participating agencies were having a sufficient supply of naloxone (n=13/17, 76.5 %), state health agency support (n=12/17, 70.6 %), and having regular training opportunities for providers (n=12/17, 70.6 %) while the top three facilitators reported by administrators working at non-participating agencies were financial assistance in providing kits (n=48/57, 84.2 %), having a sufficient supply of naloxone kits (n=48/57, 84.2 %), and support from MDHHS (n=37/57, 64.9 %).

**Table 4:** Barriers and Facilitators for Participating in the Naloxone Leave Behind Program – Participating Agency Administrators versus Non-Participating Agency Administrators.

**Question**		**Participating**				**Non-Participating**		
		**Response**	**N**	**%**		**Response**	**N**	**%**
Which of the following are barriers for participating in the leave behind program? select all that apply (Participating N=13, Not-Participating N=54)		Convincing providers to leave behind naloxone	8	61.5 %		Stocking naloxone kits	38	70.4 %
		Training providers on how to code leaving behind a naloxone kit	6	46.2 %		Financial challenge	31	57.4 %
		Provider stigma against naloxone	5	38.5 %		Convincing providers to leave behind naloxone	25	46.3 %
		Community stigma	5	38.5 %		Provider stigma against naloxone	22	40.7 %
		Convincing providers to take leave behind naloxone training	2	15.4 %		Training providers on how to code leaving behind a naloxone kit	21	38.9 %
		Stocking naloxone kits	2	15.4 %		Convincing providers to take leave behind naloxone training	21	38.9 %
		Unable to generate internal awareness of naloxone leave behind program participation	2	15.4 %		Administrative challenge	19	35.2 %
						Community stigma	18	33.3 %
						Unable to generate internal awareness of naloxone leave behind program participation	10	18.5 %
Which of the following are facilitators for participating in the leave behind program? select all that apply (Participating N=17, Not-Participating N=57)		Having a sufficient supply of naloxone kits	13	76.5 %		Financial assistance in providing kits	48	84.2 %
		Support from the MDHHS	12	70.6 %		Having a sufficient supply of naloxone kits	48	84.2 %
		Regular training opportunities for providers	12	70.6 %		Support from the MDHHS	37	64.9 %
		Supportive staff	11	64.7 %		Regular training opportunities for providers	36	63.2 %
		Local government support for naloxone kit provision	11	64.7 %		Local government support for naloxone kit provision	35	61.4 %
		Community support for naloxone kit provision	10	58.8 %		Supportive staff	33	57.9 %
		Financial assistance in providing kits	8	47.1 %		Community support for naloxone kit provision	26	45.6 %

Notes: Author’s analysis of survey response data. “Participating” reflects responses from emergency medical service program administrators whose Medical Control Authority regions were participating in the Naloxone Leave Behind program. “Non-participating” reflects responses from emergency medical service program administrators whose Medical Control Authority regions were not participating in the Naloxone Leave Behind program.

## 4 Discussion

### 4.1 Summary of findings

Our study finds that EMS field providers and administrators in Michigan, USA were more likely to agree than disagree that the NLB program has the potential to reduce fatal overdoses and that EMS plays an important role in expanding naloxone access. However, there were still some field providers and administrators who were concerned that expanding naloxone through the NLB program could lead to unintended consequences (e.g., increased drug use and reduced treatment use). We also find that participation in the program was also low among survey participants; only about a third of EMS field providers stated that they had been trained to leave behind naloxone, and only 25 % of field providers trained to leave behind naloxone and had an overdose encounter in the last month left behind a kit. Finally, our results demonstrate administrator perceptions regarding the administrative challenge of program participation were mixed and varied by program participation status.

Our findings ultimately add to the existing literature on NLB programs by being the first to evaluate perceptions in the Michigan NLB program. Michigan is unique to other locations where NLB programs were previously studied (e.g., Tennessee and San Francisco) given its more decentralized EMS regulation (using MCAs). Michigan also has significant heterogeneity in overdoses geographically with considerable racial and ethnic disparities in overdose rates and naloxone access, driving the importance of EMS NLB programs as an opportunity to increase naloxone access (Michigan Department of Health and Human Services, 2023b). Therefore, the results may be relevant to other states with decentralized EMS regulatory authorities facing disparities in naloxone access.

Our study took place during the fentanyl-era of the opioid overdose crisis, where fentanyl and other adulterants in the drug supply have contributed to increased overdose mortality and exacerbated the need for rapid public health response. Our study is also one of the only studies evaluating a NLB program implemented after the peak of the COVID-19 pandemic. Our findings may be less impacted by pandemic era disruptions compared to findings from other studies evaluating programs implemented early in the COVID-19 pandemic.

In light of all findings, we provide recommendations to increase program adoption below.

### 4.2 Implications for policy and practice

First, educating providers on the benefits of leaving behind naloxone and the NLB program’s objectives during training and education may increase the intensity of participation by individual EMS field providers and support among EMS administrators. Due to concerns by EMS administrators that some of their field providers were biased against people who use drugs, training should incorporate anti-stigma modules. To combat stigma against people who use drugs, EMS leaders could partner with local harm reduction service providers, drug users unions, or community advisory boards to provide education to EMS about the lived experiences of people who use drugs. Further, training should address providers' direct concerns about leaving behind naloxone. Educational strategies should emphasize addressing concerns that NLB could encourage more drug use or reduce the incentive for people to receive treatment. Current scientific evidence shows no relationship between expanding naloxone access and drug use (Colledge-Frisby et al., 2023, Binswanger et al., 2022) and that NLB programs likely increase drug treatment initiation (Scharf et al., 2020). Previous studies that identified that naloxone access increased drug use (Doleac and Mukherjee, 2016, Packham, 2019) have been found to have serious methodological issues with unreliable findings (Frank et al., 2018, Lambdin et al., 2023).

Second, addressing experienced and perceived barriers to participation can inspire greater involvement by EMS agencies. For example, informing non-participating agencies about the free provision of naloxone kits could make them more likely to participate, given that non-participating agencies perceived a potential financial challenge of participating in the NLB program and stocking kits even though kits are provided for free. Stocking kits was also a concern for administrators at participating agencies, so enabling sufficient ease of access to naloxone kits among participating agencies may increase naloxone distribution rates. This includes streamlining the naloxone procurement process, ensuring timely delivery of kits to providers, and minimizing related administrative challenge with acquiring kits.

Third, research regarding the impact of naloxone leave behind on EMS operations is warranted. Respondents felt that NLB would not reduce 911 calls or free up EMS field providers to respond to other emergencies. However, if NLB programs were known to be effective in reducing the operational challenge of EMS field providers, this could be used in messaging during training and other communications to encourage broader adoption.

Fourth, greater clarity around legal repercussions resulting from NLB is needed to mitigate the concerns of administrators and field providers. In Michigan, there are no legal repercussions to EMS field providers who administer or disseminate naloxone. This is true even if the administration or dissemination results in harm to the individual, so long as the administration had good faith intent (Michigan Legislature, 2014). Dispelling concerns that there could be legal action taken against field providers who leave behind naloxone may help increase adoption.

Finally, future research is needed to link changes in perceptions by EMS field providers and administrators to changes in naloxone distribution rates. Understanding which perceptions are most closely related to optimal practice patterns will be important when considering program improvement in Michigan and other states.

### 4.3 Limitations

There are several limitations to this study. Desirability bias might influence EMS field providers to be more supportive of the program and leave behind naloxone in general. Moreover, EMS field providers and administrators willing to take the survey might feel more positively or negatively toward the program than non-respondents (i.e., respondents may have more extreme opinions driving their selection into responding to the survey). Given the low response rate and lack of a well-defined sampling frame, we consider our respondents to comprise a convenience sample. Together, responses may indicate less bias against the program than a more generalizable sample without selection into participation, potentially making our findings around program benefits and enthusiasm less conservative. Respondents in the sample also tended to be highly experienced (i.e., licensed 10+ years), and their beliefs may differ from those who are younger or less experienced. Results are also specific to this program and the state of Michigan and may not generalize to other states that have different administrative processes and program designs. Evaluations in other states and programs are needed to understand how local circumstances impact perspectives on naloxone leave behind.

## 5 Conclusion

The NLB program in Michigan is a promising initiative to expand naloxone access in the state. EMS field providers and administrators in Michigan generally supported the NLB program and recognized its potential to reduce fatal overdoses, but some providers and administrators were concerned about unintended consequences and administrative challenge. MDHHS and operators of similar programs can further support EMS in leaving behind naloxone by providing additional education around the benefits of participation that address common concerns of NLB, encouraging closer collaboration between EMS field providers to optimize delivery and reporting practices, and streamlining and promoting awareness of the naloxone procurement process to prospective participants. The knowledge gained from the perspectives of EMS field providers and administrators may be useful to inform program development in other US states and around the world.

## Financial support

This project was funded through a grant from 10.13039/100015283Bloomberg Philanthropies Grant ID: 2021-97089. The funding organization had no role in the design and conduct of the study; collection, management, analysis, and interpretation of the data; preparation, review, or approval of the manuscript; and decision to submit the manuscript for publication.

## CRediT authorship contribution statement

**Hridika Shah:** Writing – review & editing, Writing – original draft, Project administration, Conceptualization. **Eric G Hulsey:** Writing – review & editing, Writing – original draft, Supervision, Conceptualization. **Samantha J Harris:** Writing – review & editing, Writing – original draft, Methodology, Conceptualization. **Olivia K Sugarman:** Writing – review & editing, Writing – original draft, Methodology, Conceptualization. **Lauren Byrne:** Writing – review & editing, Writing – original draft, Methodology, Conceptualization. **Brendan Saloner:** Writing – review & editing, Writing – original draft, Supervision, Methodology, Conceptualization. **Jason B Gibbons:** Writing – review & editing, Writing – original draft, Methodology, Formal analysis, Conceptualization. **Anthony Pantaleo:** Writing – review & editing, Writing – original draft, Supervision, Resources, Data curation, Conceptualization. **Emily Bergquist:** Writing – review & editing, Writing – original draft, Resources, Data curation, Conceptualization. **Julie Rwan:** Writing – review & editing, Writing – original draft, Supervision, Conceptualization. **Esther Rosner:** Writing – review & editing, Writing – original draft, Supervision, Conceptualization.

## Declaration of Competing Interest

The views, opinions, and findings in this article are those of the authors. Findings and interpretations do not represent official opinions of the Michigan Department of Health and Human Services. The study was funded in part by Bloomberg Philanthropies. The authors have no conflicts to report.

## Figures and Tables

**Appendix Table 1:** Perceptions of Naloxone and Naloxone Leave Behind – EMS Employees versus Administrators

**Question Description**	**EMS Administrator** **Agree**		**EMS Field-Provider** **Agree**		**P-Value**
	**N**	**%**	**N**	**%**	
**What are your perceptions about Naloxone in general?**					
Naloxone increases the rate of overdose because of a lack of fear about fatal overdose	25	30.9 %	109	37.8 %	0.248
Naloxone condones drug use	20	24.7 %	94	32.6 %	0.171
Naloxone does not address the problem of addiction	65	80.2 %	232	80.6 %	0.951
Naloxone should only be administered by medical professionals	10	12.3 %	49	17.0 %	0.311
**What are your perceptions about leaving behind Naloxone and the role of EMS?**					
EMS could have a significant public health impact by leaving behind Naloxone	41	50.6 %	164	56.9 %	0.311
EMS have a role to play in overdose prevention	29	35.8 %	136	47.2 %	0.068
Leaving behind Naloxone frees up EMS to respond to other emergencies	7	8.6 %	35	12.2 %	0.379
Leaving behind Naloxone allows EMS to do more for their community	43	53.1 %	154	53.5 %	0.951
Leaving behind naloxone is helpful for any drug overdose (e.g., stimulants)	8	10.0 %	54	18.8 %	0.063
Leaving behind naloxone would save lives	47	58.0 %	174	60.6 %	0.673
Leaving behind naloxone will encourage people to use even more opioids	24	29.6 %	104	36.2 %	0.270
Leaving behind naloxone is a waste of time and money	10	12.3 %	56	19.5 %	0.138
**What are your thoughts or perceptions of your EMS agency’s attitudes and resources to support a leave-behind naloxone program?**					
My EMS agency believes that leaving behind Naloxone can help save lives	46	56.8 %	124	43.1 %	0.028
My EMS agency encourages us regularly to leave behind Naloxone	15	18.8 %	62	21.8 %	0.561
My EMS agency has been supportive of training providers to leave-behind Naloxone	24	29.6 %	100	34.8 %	0.381
There is adequate support from my EMS agency to expand program participation	32	39.5 %	109	37.8 %	0.786
**What concerns do you have about an EMS-based leave-behind Naloxone program?**					
I am concerned about individuals managing the side effects of naloxone on their own	34	42.0 %	138	48.1 %	0.331
I am concerned that leaving behind naloxone will lead to unintended consequences	27	33.3 %	89	31.0 %	0.691
I am concerned that people who receive naloxone will be less likely to seek treatment	42	51.9 %	159	55.2 %	0.592
**Leaving behind Naloxone to family friends or bystanders or people who overdose will be effective in …**					
Increasing utilization of drug treatment programs	8	9.9 %	42	14.7 %	0.261
Reducing 911 calls	6	7.4 %	58	20.4 %	0.007
Reducing drug overdose events	20	25.0 %	83	29.1 %	0.469
Reducing drug use	2	2.5 %	17	6.0 %	0.209
Reducing overdose deaths	56	69.1 %	185	64.7 %	0.456

Notes: Author’s analysis of survey response data. Agree reflects agreement aggregated from “Agree” and “Strongly Agree” survey responses. Responses are compared between emergency medical service administrators and field providers. P-values are used to identify statistical significant differences in responses by administrators and field providers.
