# Supplementary material for: Perceptions of a naloxone leave behind program among emergency medical services personnel in Michigan, USA
Source: Drug Alcohol Depend Rep. 2024 Aug 14;12:100273. doi: 10.1016/j.dadr.2024.100273 (PMC11387810; doi:10.1016/j.dadr.2024.100273)
Supplement: Supplementary file 1 — Supplementary material [file mmc1.pdf]

Naloxone Leave Behind - Final

Start of Block: eConsent

Consent
Emergency Medical Service Naloxone Leave Behind Evaluation
Survey Consent Form

We are inviting you to participate in an electronic survey about the new Naloxone Leave Behind program as an Emergency Medical Service (EMS) affiliate in Michigan. This survey will take about ten minutes.

As part of this survey, we will ask you for your opinions about the new Naloxone Leave Behind program for EMS providers in Michigan. Specifically, we will ask you about your thoughts regarding the provision of naloxone kits to people who use drugs following an EMS overdose encounter. This will include questions about your perceptions of the program's efficacy and effectiveness in reducing future overdoses. Your answers will help us understand how this program is being perceived by EMS affiliates and agencies participating in the program and inform the Michigan Department of Health and Human Services (MDHHS) programming.

None of the questions here are personal or private, and all responses will be confidential (no personally identifiable information will be included with your responses). If you do not want to answer any question, you may skip that question. If at any point, you decide you do not want to participate in the study, you may end your participation in the study at any time by closing the survey. We will keep the information you provide safe and private. We will not ask for identifying information.

Survey participants will be entered into a raffle to receive one of 20 gift cards, each worth $25 for completing the survey.

Clicking "Proceed to Survey" below means that you have reviewed the information in this form, and you agree to join the study. You will not give up any legal rights by consenting to participate in this study. Do you wish to complete the survey?

○ Yes, proceed to survey. (1)

○ No, I do not wish to complete this survey. (2)

Skip To: End of Survey If Emergency Medical Service Naloxone Leave Behind Evaluation   Survey Consent Form We are inviting... = No, I do not wish to complete this survey.

End of Block: eConsent

Start of Block: Employment Information

X→

Q1 Are you currently employed or contracted by an EMS agency?

◯ Yes (1)

◯ No (2)

X→

Q2 What is your primary Medical Control Authority (MCA)?

▼ Allegan County MCA (1) ... Wayne County MCA (59)

Display This Question:

If What is your primary Medical Control Authority (MCA)? = Allegan County MCA

And Are you currently employed or contracted by an EMS agency? = Yes

X→

A1 What is the name of the primary EMS agency you work for?

▼ BORGESS MEDICAL CENTER DBA PLAINWELL AREA EMS (1) ... WAYLAND AREA EMERGENCY MEDICAL SERVICES (10)

Display This Question:

If What is your primary Medical Control Authority (MCA)? = Arenac County MCA

And Are you currently employed or contracted by an EMS agency? = Yes

X→

A2 What is the name of the primary EMS agency you work for?

▼ MOBILE MEDICAL RESPONSE INC (1) ... MOBILE MEDICAL RESPONSE INC (1)

Display This Question:

If What is your primary Medical Control Authority (MCA)? = Baraga County MCA

And Are you currently employed or contracted by an EMS agency? = Yes

X→

A3 What is the name of the primary EMS agency you work for?

▼ BAY AMBULANCE INC (1) ... UP HEALTH SYSTEM EMS-WEST (4)

Display This Question:

If What is your primary Medical Control Authority (MCA)? = Barry County MCA

And Are you currently employed or contracted by an EMS agency? = Yes

X→

A4 What is the name of the primary EMS agency you work for?

▼ BELLEVUE COMMUNITY FIRE DEPARTMENT (1) ... WOODLAND TOWNSHIP FIRE DEPARTMENT (14)

Display This Question:

If What is your primary Medical Control Authority (MCA)? = Bay Area MCA

And Are you currently employed or contracted by an EMS agency? = Yes

X→

A5 What is the name of the primary EMS agency you work for?

▼ AURORA MEDICAL CENTER-BAY AREA (1) ... MID COUNTY RESCUE SQUAD INC (4)

Display This Question:

If What is your primary Medical Control Authority (MCA)? = Bay County MCA

And Are you currently employed or contracted by an EMS agency? = Yes

A6 What is the name of the primary EMS agency you work for?

▼ AKRON/COLUMBIA/WISNER TOWNSHIP (1) ... PORTSMOUTH TOWNSHIP FIRE DEPARTMENT (19)

Display This Question:

If What is your primary Medical Control Authority (MCA)? = Berrien County MCA

And Are you currently employed or contracted by an EMS agency? = Yes

A7 What is the name of the primary EMS agency you work for?

▼ BRIDGMAN FIRE/RESCUE (1) ... WATERVLIET FIRE DEPARTMENT (14)

Display This Question:

If What is your primary Medical Control Authority (MCA)? = Branch County MCA

And Are you currently employed or contracted by an EMS agency? = Yes

A8 What is the name of the primary EMS agency you work for?

▼ BRONSON FIRE DEPARTMENT (1) ... TEKONSHA FIRE DEPARTMENT (8)

Display This Question:

If What is your primary Medical Control Authority (MCA)? = Calhoun County MCA

And Are you currently employed or contracted by an EMS agency? = Yes

A9 What is the name of the primary EMS agency you work for?

▼ ALBION TOWNSHIP FIRE DEPARTMENT (1) ... VETERANS AFFAIRS (DEPARTMENT OF) FIRE DEPARTMENT (22)

Display This Question:

If What is your primary Medical Control Authority (MCA)? = Cass County MCA

And Are you currently employed or contracted by an EMS agency? = Yes

X→

A10 What is the name of the primary EMS agency you work for?

▼ COLOMA EMERGENCY AMBULANCE INC DBA PRIDE CARE (1) ... SEPSA FIRE AUTHORITY (7)

Display This Question:

If What is your primary Medical Control Authority (MCA)? = Clare County MCA

And Are you currently employed or contracted by an EMS agency? = Yes

X→

A11 What is the name of the primary EMS agency you work for?

▼ GARFIELD TOWNSHIP FIRE DEPARTMENT (CLARE COUNTY) (1) ... SURREY TOWNSHIP FIRE DEPARTMENT (6)

Display This Question:

If What is your primary Medical Control Authority (MCA)? = Delta County MCA

And Are you currently employed or contracted by an EMS agency? = Yes

X→

A12 What is the name of the primary EMS agency you work for?

▼ BALDWIN TOWNSHIP VOLUNTEER FIRE DEPARTMENT/FIRST RESPONDERS (1) ... TRI-STAR EMS INC (6)

Display This Question:

If What is your primary Medical Control Authority (MCA)? = Detroit East MCA

And Are you currently employed or contracted by an EMS agency? = Yes

X→

A13 What is the name of the primary EMS agency you work for?

▼ DETROIT FIRE DEPARTMENT EMS (1) ... UNIVERSAL-MACOMB AMBULANCE SERVICE (12)

Display This Question:

If What is your primary Medical Control Authority (MCA)? = Dickinson County MCA

And Are you currently employed or contracted by an EMS agency? = Yes

X→

A14 What is the name of the primary EMS agency you work for?

▼ FAITHORN FIRE AND RESCUE (1) ... VERSO CORPORATION (9)

Display This Question:

If What is your primary Medical Control Authority (MCA)? = Eastern UP MCA

And Are you currently employed or contracted by an EMS agency? = Yes

X→

A15 What is the name of the primary EMS agency you work for?

▼ BAY MILLS EMERGENCY CONNECTION (1) ... SUGAR ISLAND AMBULANCE CORPS (8)

Display This Question:

If What is your primary Medical Control Authority (MCA)? = Genesee County MCA

And Are you currently employed or contracted by an EMS agency? = Yes

X→

A16 What is the name of the primary EMS agency you work for?

▼ ARGENTINE FIRE & RESCUE (1) ... TWIN TOWNSHIP AMBULANCE INC (21)

Display This Question:

If What is your primary Medical Control Authority (MCA)? = Gogebic/Ontonagon/Iron MCA

And Are you currently employed or contracted by an EMS agency? = Yes

X→

A17 What is the name of the primary EMS agency you work for?

▼ ASPIRUS WAUSAU HOSPITAL INC (ASPIRUS MEDEVAC) (1) ... WATERSMEET VOLUNTEER FIRE DEPARTMENT (4)

Display This Question:

If What is your primary Medical Control Authority (MCA)? = Gratiot County MCA

And Are you currently employed or contracted by an EMS agency? = Yes

X→

A18 What is the name of the primary EMS agency you work for?

▼ ALMA DISTRICT FIRE & RESCUE (1) ... MOBILE MEDICAL RESPONSE OF GRATIOT COUNTY (7)

Display This Question:

If What is your primary Medical Control Authority (MCA)? = Hillsdale County MCA

And Are you currently employed or contracted by an EMS agency? = Yes

X→

A19 What is the name of the primary EMS agency you work for?

▼ ADDISON FIRE DEPARTMENT (1) ... WRIGHT TOWNSHIP AMBULANCE SERVICE (HILLSDALE COUNTY) (15)

Display This Question:

If What is your primary Medical Control Authority (MCA)? = Huron County MCA

And Are you currently employed or contracted by an EMS agency? = Yes

X→

A20 What is the name of the primary EMS agency you work for?

▼ CENTRAL HURON AMBULANCE SERVICE ASSOCIATION (1) ... SIGEL TOWNSHIP FIRE DEPARTMENT (7)

Display This Question:

If What is your primary Medical Control Authority (MCA)? = Ionia County MCA

And Are you currently employed or contracted by an EMS agency? = Yes

X→

A21 What is the name of the primary EMS agency you work for?

▼ BELDING FIRE DEPARTMENT (1) ... SUNFIELD FIRE DEPARTMENT (16)

Display This Question:

If What is your primary Medical Control Authority (MCA)? = Iosco County MCA

And Are you currently employed or contracted by an EMS agency? = Yes

X→

A22 What is the name of the primary EMS agency you work for?

▼ EAST TAWAS FIRE DEPARTMENT (1) ... TAWAS CITY FIRE DEPARTMENT (7)

Display This Question:

If What is your primary Medical Control Authority (MCA)? = Isabella County MCA

And Are you currently employed or contracted by an EMS agency? = Yes

X→

A23 What is the name of the primary EMS agency you work for?

▼ COLEMAN COMMUNITY FIRE DEPARTMENT (1) ... SURREY TOWNSHIP FIRE DEPARTMENT (9)

Display This Question:

If What is your primary Medical Control Authority (MCA)? = Jackson County MCA

And Are you currently employed or contracted by an EMS agency? = Yes

A24 What is the name of the primary EMS agency you work for?

▼ ADDISON FIRE DEPARTMENT (1) ... SURVIVAL FLIGHT (23)

Display This Question:

If What is your primary Medical Control Authority (MCA)? = Kalamazoo County MCA

And Are you currently employed or contracted by an EMS agency? = Yes

A25 What is the name of the primary EMS agency you work for?

▼ ALAMO FIRE DEPARTMENT (1) ... WEST MICHIGAN AIR CARE (20)

Display This Question:

If What is your primary Medical Control Authority (MCA)? = Kent County MCA

And Are you currently employed or contracted by an EMS agency? = Yes

A26 What is the name of the primary EMS agency you work for?

▼ ADA TOWNSHIP FIRE DEPARTMENT (1) ... WYOMING (CITY OF) FIRE DEPARTMENT (39)

Michigan Department of Health & Human Services

Display This Question:

If What is your primary Medical Control Authority (MCA)? = Keweenaw MCA

And Are you currently employed or contracted by an EMS agency? = Yes

X→

A27 What is the name of the primary EMS agency you work for?

▼ ADAMS TOWNSHIP VOLUNTEER FIRE DEPARTMENT (1) ... STANTON TOWNSHIP (11)

Display This Question:

If What is your primary Medical Control Authority (MCA)? = Lapeer County MCA

And Are you currently employed or contracted by an EMS agency? = Yes

X→

A28 What is the name of the primary EMS agency you work for?

▼ ALMONT MEDICAL FIRST RESPONDERS (1) ... PATRIOT AMBULANCE SERVICE INC (13)

Display This Question:

If What is your primary Medical Control Authority (MCA)? = Lenawee County MCA

And Are you currently employed or contracted by an EMS agency? = Yes

X→

A29 What is the name of the primary EMS agency you work for?

▼ ADDISON FIRE DEPARTMENT (1) ... TECUMSEH FIRE DEPARTMENT (23)

Display This Question:

If What is your primary Medical Control Authority (MCA)? = Luce County MCA

And Are you currently employed or contracted by an EMS agency? = Yes

X→

A30 What is the name of the primary EMS agency you work for?

▼ GARFIELD TOWNSHIP AMBULANCE SERVICE (MACKINAC COUNTY) (1) ... WHITEFISH TOWNSHIP EMS (6)

Display This Question:

If What is your primary Medical Control Authority (MCA)? = Macomb County MCA

And Are you currently employed or contracted by an EMS agency? = Yes

A31 What is the name of the primary EMS agency you work for?

▼ ARMADA FIRE DEPARTMENT EMS (1) ... WASHINGTON CHARTER TOWNSHIP FIRE DEPARTMENT (30)

Display This Question:

If What is your primary Medical Control Authority (MCA)? = Manistee County MCA

And Are you currently employed or contracted by an EMS agency? = Yes

A32 What is the name of the primary EMS agency you work for?

▼ ARCADIA TOWNSHIP FIRE DEPARTMENT (1) ... THOMPSONVILLE AMBULANCE SERVICE (14)

Display This Question:

If What is your primary Medical Control Authority (MCA)? = Marquette-Alger MCA

And Are you currently employed or contracted by an EMS agency? = Yes

A33 What is the name of the primary EMS agency you work for?

▼ ALGER COUNTY AMBULANCE SERVICE (1) ... UP HEALTH SYSTEM EMS-WEST (17)

Display This Question:

If What is your primary Medical Control Authority (MCA)? = Mason County MCA

And Are you currently employed or contracted by an EMS agency? = Yes

A34 What is the name of the primary EMS agency you work for?

▼ BRANCH TOWNSHIP FIRE DEPARTMENT (1) ... WESTERN MASON COUNTY FIRE DISTRICT AUTHORITY (10)

Display This Question:

If What is your primary Medical Control Authority (MCA)? = Midland-Gladwin County MCA

And Are you currently employed or contracted by an EMS agency? = Yes

A35 What is the name of the primary EMS agency you work for?

▼ BEAVERTON AREA FIRE DEPARTMENT (1) ... WILDWOOD FIRE DEPARTMENT (22)

Display This Question:

If What is your primary Medical Control Authority (MCA)? = Monroe County MCA

And Are you currently employed or contracted by an EMS agency? = Yes

A36 What is the name of the primary EMS agency you work for?

▼ ASH TOWNSHIP FIRE DEPARTMENT (1) ... WHITEFORD TOWNSHIP VOLUNTEER FIRE DEPARTMENT (23)

Display This Question:

If What is your primary Medical Control Authority (MCA)? = Montcalm County MCA

And Are you currently employed or contracted by an EMS agency? = Yes

A37 What is the name of the primary EMS agency you work for?

▼ MONTCALM (COUNTY OF) (1) ... MONTCALM (COUNTY OF) (1)

Display This Question:

If What is your primary Medical Control Authority (MCA)? = Muskegon County MCA

And Are you currently employed or contracted by an EMS agency? = Yes

X→

A38 What is the name of the primary EMS agency you work for?

▼ BLUE LAKE TOWNSHIP FIRE DEPARTMENT (MUSKEGON COUNTY) (1) ... WHITE LAKE FIRE AUTHORITY (MUSKEGON COUNTY) (17)

Display This Question:

If What is your primary Medical Control Authority (MCA)? = Newaygo County MCA

And Are you currently employed or contracted by an EMS agency? = Yes

X→

A39 What is the name of the primary EMS agency you work for?

▼ ASHLAND & GRANT FIRE DISTRICT (1) ... WALKERVILLE AREA FIRE & RESCUE (10)

Display This Question:

If What is your primary Medical Control Authority (MCA)? = North Central MCA

And Are you currently employed or contracted by an EMS agency? = Yes

X→

A40 What is the name of the primary EMS agency you work for?

▼ BEAVER CREEK FIRE DEPARTMENT (1) ... SOUTH BRANCH TOWNSHIP (13)

Display This Question:

If What is your primary Medical Control Authority (MCA)? = North Central Michigan MCA

And Are you currently employed or contracted by an EMS agency? = Yes

A41 What is the name of the primary EMS agency you work for?

▼ BIG RAPIDS (CITY OF) FIRE DEPARTMENT (1) ... WHEATLAND TOWNSHIP FIRE & RESCUE (23)

Display This Question:

If What is your primary Medical Control Authority (MCA)? = Northeast Michigan MCA

And Are you currently employed or contracted by an EMS agency? = Yes

A42 What is the name of the primary EMS agency you work for?

▼ ALCONA COUNTY EMS (1) ... TRI TOWNSHIP AMBULANCE SERVICE (MONTMORENCY COUNTY) (26)

Display This Question:

If What is your primary Medical Control Authority (MCA)? = Northern Michigan MCA

And Are you currently employed or contracted by an EMS agency? = Yes

A43 What is the name of the primary EMS agency you work for?

▼ BANKS TOWNSHIP FIRE & RESCUE (1) ... RFC FIRE/RESCUE (21)

Display This Question:

If What is your primary Medical Control Authority (MCA)? = Northwest Regional MCA

And Are you currently employed or contracted by an EMS agency? = Yes

A44 What is the name of the primary EMS agency you work for?

▼ ALMIRA TOWNSHIP (1) ... WHITEWATER TOWNSHIP FIRE DEPARTMENT (44)

Display This Question:

If What is your primary Medical Control Authority (MCA)? = Oakland County MCA

And Are you currently employed or contracted by an EMS agency? = Yes

X→

A45 What is the name of the primary EMS agency you work for?

▼ ADDISON TOWNSHIP FIRE DEPARTMENT (1) ... WIXOM FIRE DEPARTMENT (49)

Display This Question:

If What is your primary Medical Control Authority (MCA)? = Oceana County MCA

And Are you currently employed or contracted by an EMS agency? = Yes

X→

A46 What is the name of the primary EMS agency you work for?

▼ FERRY TOWNSHIP FIRE DEPARTMENT (1) ... WALKERVILLE AREA FIRE & RESCUE (7)

Display This Question:

If What is your primary Medical Control Authority (MCA)? = Ogemaw County MCA

And Are you currently employed or contracted by an EMS agency? = Yes

X→

A47 What is the name of the primary EMS agency you work for?

▼ DENTON TOWNSHIP AMBULANCE SERVICE (1) ... RICHFIELD TOWNSHIP (6)

Display This Question:

If What is your primary Medical Control Authority (MCA)? = Otsego County MCA

And Are you currently employed or contracted by an EMS agency? = Yes

X→

A48 What is the name of the primary EMS agency you work for?

▼ Albert Township Fire Department (1) ... OTSEGO LAKE TOWNSHIP FIRE DEPARTMENT (6)

Display This Question:

If What is your primary Medical Control Authority (MCA)? = Ottawa County MCA

And Are you currently employed or contracted by an EMS agency? = Yes

X→

A49 What is the name of the primary EMS agency you work for?

▼ ALLENDALE FIRE DEPARTMENT (1) ... ZEELAND FIRE RESCUE (28)

Display This Question:

If What is your primary Medical Control Authority (MCA)? = Saginaw/Tuscola MCA

And Are you currently employed or contracted by an EMS agency? = Yes

X→

A50 What is the name of the primary EMS agency you work for?

▼ AIR METHODS / ROCKY MOUNTAIN HOLDINGS LLC DBA LIFE NET OF MICHIGAN (1) ... ZILWAUKEE (CITY OF) FIRE DEPARTMENT (27)

Display This Question:

If What is your primary Medical Control Authority (MCA)? = Saint Clair County MCA

And Are you currently employed or contracted by an EMS agency? = Yes

X→

A51 What is the name of the primary EMS agency you work for?

▼ ALGONAC FIRE DEPARTMENT (1) ... TRI-HOSPITAL EMS CORPORATION (22)

Display This Question:

If What is your primary Medical Control Authority (MCA)? = Saint Joseph County MCA

And Are you currently employed or contracted by an EMS agency? = Yes

Q52 What is the name of the primary EMS agency you work for?

▼ BURR OAK COMMUNITY FIRE DEPARTMENT (1) ... TRI-TOWNSHIP FIRE DEPARTMENT (SAINT JOSEPH COUNTY) (11)

Display This Question:

If What is your primary Medical Control Authority (MCA)? = Sanilac County MCA

And Are you currently employed or contracted by an EMS agency? = Yes

Q53 What is the name of the primary EMS agency you work for?

▼ ARGYLE FIRE DEPARTMENT (1) ... TRI-HOSPITAL EMS CORPORATION (15)

Display This Question:

If What is your primary Medical Control Authority (MCA)? = Schoolcraft County MCA

And Are you currently employed or contracted by an EMS agency? = Yes

A54 What is the name of the primary EMS agency you work for?

▼ DOYLE TOWNSHIP FIRST RESPONDERS (1) ... RAMPART EMS (7)

Display This Question:

If What is your primary Medical Control Authority (MCA)? = Shiawassee County MCA

And Are you currently employed or contracted by an EMS agency? = Yes

A55 What is the name of the primary EMS agency you work for?

▼ CORUNNA AREA AMBULANCE SERVICE (1) ... TWIN TOWNSHIP AMBULANCE INC (9)

Display This Question:

If What is your primary Medical Control Authority (MCA)? = Tri-County MCA

And Are you currently employed or contracted by an EMS agency? = Yes

A56 What is the name of the primary EMS agency you work for?

▼ BATH TOWNSHIP FIRE & RESCUE DEPARTMENT (1) ... WINDSOR TOWNSHIP EMERGENCY SERVICES (46)

Display This Question:

If What is your primary Medical Control Authority (MCA)? = Van Buren County MCA

And Are you currently employed or contracted by an EMS agency? = Yes

A57 What is the name of the primary EMS agency you work for?

▼ BANGOR COMMUNITY FIRE DEPARTMENT (VAN BUREN COUNTY) (1) ... VBEMS INC (14)

Display This Question:

If What is your primary Medical Control Authority (MCA)? = Washtenaw/Livingston MCA

And Are you currently employed or contracted by an EMS agency? = Yes

A58 What is the name of the primary EMS agency you work for?

▼ ANN ARBOR FIRE DEPARTMENT (1) ... YPSILANTI TOWNSHIP FIRE DEPARTMENT (30)

Display This Question:

If What is your primary Medical Control Authority (MCA)? = Wayne County MCA

And Are you currently employed or contracted by an EMS agency? = Yes

A59 What is the name of the primary EMS agency you work for?

▼ ALLEN PARK FIRE DEPARTMENT (1) ... WYANDOTTE FIRE DEPARTMENT (43)

Q4 What is your primary role?

▼ Full-time field provider (1) ... Medical director (10)

Q5 For how many years have you been working in this role?

▼ < 1 year (1) ... >= 10 years (5)

Q6 What is your current license level?

▼ Medical first responder (1) ... None of the above (11)

Display This Question:

If What is your current license level? != None of the above

Q7 How many years ago did you receive your EMS license?

▼ < 1 year (1) ... I do not have an EMS license (6)

End of Block: Employment Information

Start of Block: Practice

Display This Question:

If Are you currently employed or contracted by an EMS agency? = Yes

And If

What is your primary role? = Full-time field provider

Or What is your primary role? = Part-time field provider

Or What is your primary role? = Paid on-call or volunteer provider

Q8 Approximately how many opioid related overdoses have you responded to in the last 30 days?

▼ 0 (1) ... > 20 (6)

Display This Question:

If Are you currently employed or contracted by an EMS agency? = Yes

And If

What is your primary role? = Full-time field provider

Or What is your primary role? = Part-time field provider

Or What is your primary role? = Paid on-call or volunteer provider

Q9 Approximately how many naloxone administrations have you performed during opioid related overdoses in the last 30 days?

▼ 0 (1) ... > 20 (6)

Display This Question:

If Are you currently employed or contracted by an EMS agency? = Yes

And If

What is your primary role? = Full-time field provider

Or What is your primary role? = Part-time field provider

Or What is your primary role? = Paid on-call or volunteer provider

X→

Q10 Approximately how many take home Naloxone kits have you provided during and overdose dispatch in the last 30 days?

▼ 0 (1) ... > 20 (6)

End of Block: Practice

Start of Block: Knowledge of Leave Behind Program

Text1 Michigan's Naloxone Leave-Behind program is a new program that includes a training program for EMTs and provides naloxone (also called Narcan) kits to leave behind when dispatched for an overdose. As part of the program, EMTs are trained to leave behind naloxone when dispatched for an overdose. The naloxone kits contain instructions for use as well as information about local substance use treatment programs.

X→

Q11 Have you heard about the Naloxone Leave Behind Program?

- Yes (1)
- No (2)
- Unsure (3)

Display This Question:

If Are you currently employed or contracted by an EMS agency? = Yes

X→

Q12 Is your EMS agency participating in the Naloxone Leave Behind Program?

- Yes (1)
- No (2)
- Unsure (3)

Skip To: Q14 If Is your EMS agency participating in the Naloxone Leave Behind Program? = Yes

---

Display This Question:

If Are you currently employed or contracted by an EMS agency? = Yes

X→

Q13 If your EMS agency is not participating in the Naloxone Leave Behind Program, would you like them to?

- Yes (1)
- No (2)
- Unsure (3)

Page Break

Q14 What are your thoughts or perceptions of your EMS agency's attitudes and resources to support a leave-behind naloxone program?

	Strongly disagree (1)	Disagree (2)	Neither agree not disagree (3)	Agree (4)	Strongly agree (5)
There is adequate support from my EMS agency to expand the use of leave-behind Naloxone. (1)	o	o	o	o	o
My EMS agency has been supportive of training providers to learn how to leave-behind Naloxone. (2)	o	o	o	o	o
My EMS agency believes that leaving behind Naloxone can help save lives. (3)	o	o	o	o	o
My EMS agency encourages us regularly to leave behind Naloxone. (4)	o	o	o	o	o

Page Break

Display This Question:

If Are you currently employed or contracted by an EMS agency? = Yes

And If

What is your primary role? = Full-time field provider

Or What is your primary role? = Part-time field provider

Or What is your primary role? = Paid on-call or volunteer provider

X→

Q15 Have you received training as part of the Naloxone Leave Behind Program?

○ Yes (1)

○ No (2)

○ Unsure (3)

Skip To: Q17 If Have you received training as part of the Naloxone Leave Behind Program? = No

Skip To: Q17 If Have you received training as part of the Naloxone Leave Behind Program? = Unsure

Page Break

Display This Question:

If Are you currently employed or contracted by an EMS agency? = Yes

And If

What is your primary role? = Full-time field provider

Or What is your primary role? = Part-time field provider

Or What is your primary role? = Paid on-call or volunteer provider

X→

Q16 If you have received training as part of the Naloxone Leave Behind Program, approximately when did you complete the training?

▼ < 3 months ago (1) ... > 2 years ago (5)

Display This Question:

If Are you currently employed or contracted by an EMS agency? = Yes

And If

What is your primary role? = Full-time field provider

Or What is your primary role? = Part-time field provider

Or What is your primary role? = Paid on-call or volunteer provider

X→

Q17 My preferred way to receive EMS training is:

▼ MI-Train/Online training (1) ... Other (5)

End of Block: Knowledge of Leave Behind Program

Start of Block: Leave Behind Training (Providers, Admin, Educators)

Display This Question:

If Are you currently employed or contracted by an EMS agency? = Yes

And If

What is your primary role? = Full-time field provider

Or What is your primary role? = Part-time field provider

Or What is your primary role? = Paid on-call or volunteer provider

X→

Q18 Regardless of whether you were trained in the Naloxone Leave Behind Program, have you otherwise been trained to provide take-home Naloxone kits?

- Yes (1)
- No (2)
- Unsure (3)

Skip To: End of Block If Regardless of whether you were trained in the Naloxone Leave Behind Program, have you otherwise b... != Yes

---

Display This Question:

If Are you currently employed or contracted by an EMS agency? = Yes

And If

What is your primary role? = Full-time field provider

Or What is your primary role? = Part-time field provider

Or What is your primary role? = Paid on-call or volunteer provider

X→

Q19 Regardless of whether you were trained in the Naloxone Leave Behind Program, how long ago did you most recently receive Naloxone administration training?

- ◯ < 3 months ago (1)
- ◯ 3-6 months ago (2)
- ◯ 6-12 months ago (3)
- ◯ 12-24 months ago (4)
- ◯ > 2 years ago (5)

Display This Question:

If Are you currently employed or contracted by an EMS agency? = Yes

And If

What is your primary role? = Agency administrator

Or What is your primary role? = Full-time EMS education

Or What is your primary role? = Part-time EMS education

X→

Q20 Has your agency provided leave behind Naloxone training to your EMS providers?

- ◯ Yes (1)
- ◯ No (2)
- ◯ Unsure (3)

Skip To: End of Block If Has your agency provided leave behind Naloxone training to your EMS providers? != Yes

---

Display This Question:

If Are you currently employed or contracted by an EMS agency? = Yes

And If

What is your primary role? = Agency administrator

Or What is your primary role? = Full-time EMS education

Or What is your primary role? = Part-time EMS education

Q21 Approximately what proportion of your providers have been trained to leave behind Naloxone?

- ○ 1%-25% (1)
- ○ 26%-50% (2)
- ○ 51%-75% (3)
- ○ 76%-100% (4)

Display This Question:

If Are you currently employed or contracted by an EMS agency? = Yes

And If

What is your primary role? = Agency administrator

Or What is your primary role? = Full-time EMS education

Or What is your primary role? = Part-time EMS education

Q22 How many times per year do you train your staff to administer Naloxone?

____

End of Block: Leave Behind Training (Providers, Admin, Educators)

Start of Block: Leave Behind Perceptions - General

Q23 We want to know more about your thoughts about naloxone programs, which are programs that aim to expand the provision of Naloxone by EMS providers to members of the public. What are your thoughts or perceptions about Naloxone **in general**?

	Strongly disagree (1)	Disagree (2)	Neither agree nor disagree (3)	Agree (4)	Strongly agree (5)
Naloxone condones drug use. (1)	○	○	○	○	○
Naloxone does not address the problem of addiction. (2)	○	○	○	○	○
Naloxone access increases the rate of overdose because of lack of fear about risk of death. (3)	○	○	○	○	○
Naloxone is a medication that should only be administered by medical professionals. (4)	○	○	○	○	○

End of Block: Leave Behind Perceptions - General

Start of Block: EMS-based leave-behind program perceptions - [program]

X→

Q24 What are your thoughts or perceptions about an EMS-based leave-behind naloxone program?

	Strongly disagree (1)	Disagree (2)	Neither agree nor disagree (3)	Agree (4)	Strongly agree (5)
Leaving behind naloxone kits to people who have just experienced an overdose for future use would save lives. (1)	○	○	○	○	○
Providing a naloxone kit for future use after a person overdoses will encourage them to use even more opioids. (2)	○	○	○	○	○
Providing leave-behind naloxone kits is a waste of time and money. (3)	○	○	○	○	○
Leaving behind naloxone is helpful for any drug overdose (e.g., stimulants) (4)	○	○	○	○	○

Page Break

Michigan Department of Health & Human Services

Q25 What are your thoughts or perceptions about an EMS-based leave-behind naloxone program and the role of EMS?

	Strongly disagree (1)	Disagree (2)	Neither agree nor disagree (3)	Agree (4)	Strongly agree (5)
EMS have a role to play in overdose prevention by leaving behind Naloxone kits. (1)	○	○	○	○	○
EMS could have a significant public health impact by providing Naloxone to people who use opioid pain medications, heroin, or other opioids. (2)	○	○	○	○	○
Leaving behind Naloxone frees up EMS to respond to other emergencies. (3)	○	○	○	○	○
Leaving behind Naloxone is a tool that allows EMS to do more for their community. (4)	○	○	○	○	○

Page Break

Q27 What concerns do you have about an EMS-based leave-behind Naloxone program?

	Strongly disagree (1)	Disagree (2)	Neither agree nor disagree (3)	Agree (4)	Strongly agree (5)
I am concerned that people who use opioids will be less likely to seek out treatment if they have access to naloxone. (1)	o	o	o	o	o
I am concerned about individuals managing the side effects of naloxone on their own. (2)	o	o	o	o	o
I am concerned that giving Naloxone kits to friends and family members of someone who has overdosed to respond to a future opioid overdose with Naloxone will lead to unintended consequences. (3)	o	o	o	o	o

Page Break

Display This Question:

If Are you currently employed or contracted by an EMS agency? = Yes

And If

What is your primary role? = Full-time field provider

Or What is your primary role? = Part-time field provider

Or What is your primary role? = Paid on-call or volunteer provider

X→

Q26a What are your thoughts and perceptions about the impact of an EMS-based leave hind Naloxone program on your work?

	Strongly disagree (1)	Disagree (2)	Neither agree nor disagree (3)	Agree (4)	Strongly agree (5)
Leaving behind naloxone kits to people who have overdosed would be an added burden to my work. (1)	○	○	○	○	○
Leaving behind naloxone is a tool that allows me to do more for the community. (2)	○	○	○	○	○
I am worried that I will be held legally liable for negative events after leaving behind Naloxone kits. (3)	○	○	○	○	○

Page Break

Display This Question:

If Are you currently employed or contracted by an EMS agency? = Yes

And What is your primary role? = Agency administrator

X→

Q26b What are your thoughts and perceptions about the impact of an EMS-based leave-behind Naloxone program on EMS agencies?

	Strongly disagree (1)	Disagree (2)	Neither agree nor disagree (3)	Agree (4)	Strongly agree (5)
Naloxone leave behind programs create significant administrative challenges for EMS agencies. (1)	o	o	o	o	o
Naloxone leave behind programs put financial stress on my EMS agency. (2)	o	o	o	o	o
Participating in the leave behind Naloxone program would add burden to my work. (3)	o	o	o	o	o
I am worried that my staff or agency will be held legally liable for negative events after leaving behind Naloxone kits. (4)	o	o	o	o	o

End of Block: EMS-based leave-behind program perceptions - [program]

Start of Block: EMS-based leave-behind program perceptions - [interpersonal/patient-level]

Q28 How effective do you think leaving behind Naloxone to **people who overdose** will be in:

	Very ineffective (1)	Somewhat ineffective (2)	Neither effective nor ineffective (3)	Somewhat effective (4)	Very effective (5)
Reducing overdose deaths (1)	○	○	○	○	○
Reducing drug overdose events (2)	○	○	○	○	○
Reducing drug use (3)	○	○	○	○	○
Reducing 911 calls (4)	○	○	○	○	○
Increasing utilization of drug treatment programs (5)	○	○	○	○	○

Page Break

Q29 How effective do you think leaving behind Naloxone to **family, friends, or bystanders** or people who overdose will be in:

	Very ineffective (1)	Somewhat ineffective (2)	Neither effective nor ineffective (3)	Somewhat effective (4)	Very effective (5)
Reducing overdose deaths (1)	○	○	○	○	○
Reducing drug overdose events (2)	○	○	○	○	○
Reducing drug use (3)	○	○	○	○	○
Reducing 911 calls (4)	○	○	○	○	○
Increasing utilization of drug treatment programs (5)	○	○	○	○	○

End of Block: EMS-based leave-behind program perceptions - [interpersonal/patient-level]

Start of Block: EMS-based leave-behind program perceptions - [provider-level]

Display This Question:

If Are you currently employed or contracted by an EMS agency? = Yes

And If

What is your primary role? = Full-time field provider

Or What is your primary role? = Part-time field provider

Or What is your primary role? = Paid on-call or volunteer provider

Q30 How comfortable and/or confident are you in providing Naloxone to people who might need it?

	Strongly disagree (1)	Disagree (2)	Neither agree nor disagree (3)	Agree (4)	Strongly agree (5)
I am comfortable in having my staff provide naloxone kits to people who need it. (1)	o	o	o	o	o
I am confident in my staff's ability to identify people who have overdosed that may benefit from having naloxone kits on hand for future use. (2)	o	o	o	o	o

End of Block: EMS-based leave-behind program perceptions - [provider-level]

Start of Block: EMS-based leave-behind program perceptions (Admin ONLY)

Display This Question:

If Are you currently employed or contracted by an EMS agency? = Yes

And What is your primary role? = Agency administrator

X→

Q31 How comfortable and/or confident are you in having your staff provide Naloxone to people who might need it?

	Strongly disagree (1)	Disagree (2)	Neither agree nor disagree (3)	Agree (4)	Strongly agree (5)
I am comfortable in having my staff provide naloxone kits to people who need it. (1)	o	o	o	o	o
I am confident in my staff's ability to identify people who have overdosed that may benefit from having naloxone kits on hand for future use. (2)	o	o	o	o	o

Display This Question:

If Are you currently employed or contracted by an EMS agency? = Yes

And What is your primary role? = Agency administrator

X→

Q32 Which of the following are barriers for participating in the leave behind program? Please select all that apply.

- ☐ Training providers on how to code leaving behind a naloxone kit (1)
- ☐ Convincing providers to leave behind naloxone (2)
- ☐ Convincing providers to take leave behind naloxone training (3)
- ☐ Stocking naloxone kits (4)
- ☐ Unable to generate internal awareness of naloxone leave behind program participation (5)
- ☐ Provider stigma against naloxone (6)
- ☐ Administrative burden (7)
- ☐ Financial burden (8)
- ☐ Community stigma (9)

Page Break

Display This Question:

If Are you currently employed or contracted by an EMS agency? = Yes

And What is your primary role? = Agency administrator

X→

Q33 Which of the following are facilitators for participating in the leave behind program? Please select all that apply.

- ☐ Supportive staff (1)
- ☐ Support from the MDHHS (2)
- ☐ Financial assistance in providing kits (3)
- ☐ Regular training opportunities for providers (4)
- ☐ Community support for naloxone kit provision (5)
- ☐ Local government support for naloxone kit provision (6)
- ☐ Having a sufficient supply of naloxone kits (7)

End of Block: EMS-based leave-behind program perceptions (Admin ONLY)

Start of Block: Demographics

X→

Age What is your age (years)?

- 18-21 (1)
- 22-25 (2)
- 26-35 (3)
- 36-45 (4)
- 46-55 (5)
- 56-65 (6)
- > 65 years (7)
- Prefer not to answer (8)

Sex What is your sex?

- Male (1)
- Female (2)
- Other (3)
- Prefer not to answer (4)

Race What is your race?

- ◯ Asian (1)
- ◯ Black or African American (2)
- ◯ Native Hawaiian or Pacific Islander (3)
- ◯ Native American/American Indian or Alaskan Native (4)
- ◯ White (5)
- ◯ Other (6)
- ◯ More than one race (7)
- ◯ Prefer not to answer (8)

Hispanic Are you of Hispanic, Latino, or Spanish origin?

- ◯ Yes (1)
- ◯ No (2)
- ◯ Prefer not to answer (3)

End of Block: Demographics

Start of Block: Email for Raflle

Q116 Thank you for your time and for completing this survey!

If you wish to be entered into the gift card giveaway for a chance to win 1 of 20, $25 gift cards, please enter your email below.

Your email will not be shared with anyone and will only be used to send you the digital gift card if you are selected in the random drawing.

____

End of Block: Email for Raflle

Start of Block: Block 12
